# Blast cells surviving acute myeloid leukemia induction therapy are in cycle with a signature of FOXM1 activity

**DOI:** 10.1186/s12885-021-08839-9

**Published:** 2021-10-28

**Authors:** Mark S. Williams, Naseer J. Basma, Fabio M. R. Amaral, Daniel H. Wiseman, Tim C. P. Somervaille

**Affiliations:** 1 Leukaemia Biology Laboratory, Cancer Research UK Manchester Institute, Oglesby Cancer Research Building, The University of Manchester, 555 Wilmslow Road, Manchester, M20 4GJ UK; 2 Epigenetics of Haematopoiesis Group, Oglesby Cancer Research Building, The University of Manchester, Manchester, M20 4GJ UK

**Keywords:** Acute myeloid leukemia, Drug resistance, FOXM1, Leukemia stem cell, Transcriptome, Quiescence

## Abstract

**Background:**

Disease relapse remains common following treatment of acute myeloid leukemia (AML) and is due to chemoresistance of leukemia cells with disease repopulating potential. To date, attempts to define the characteristics of in vivo resistant blasts have focused on comparisons between leukemic cells at presentation and relapse. However, further treatment responses are often seen following relapse, suggesting that most blasts remain chemosensitive. We sought to characterise in vivo chemoresistant blasts by studying the transcriptional and genetic features of blasts from before and shortly after induction chemotherapy using paired samples from six patients with primary refractory AML.

**Methods:**

Leukemic blasts were isolated by fluorescence-activated cell sorting. Fluorescence in situ hybridization (FISH), targeted genetic sequencing and detailed immunophenotypic analysis were used to confirm that sorted cells were leukemic. Sorted blasts were subjected to RNA sequencing. Lentiviral vectors expressing short hairpin RNAs were used to assess the effect of *FOXM1* knockdown on colony forming capacity, proliferative capacity and apoptosis in cell lines, primary AML cells and CD34+ cells from healthy donors.

**Results:**

Molecular genetic analysis revealed early clonal selection occurring after induction chemotherapy. Immunophenotypic characterisation found leukemia-associated immunophenotypes in all cases that persisted following treatment. Despite the genetic heterogeneity of the leukemias studied, transcriptional analysis found concerted changes in gene expression in resistant blasts. Remarkably, the gene expression signature suggested that post-chemotherapy blasts were more proliferative than those at presentation. Resistant blasts also appeared less differentiated and expressed leukemia stem cell (LSC) maintenance genes. However, the proportion of immunophenotypically defined LSCs appeared to decrease following treatment, with implications for the targeting of these cells on the basis of cell surface antigen expression. The refractory gene signature was highly enriched with targets of the transcription factor FOXM1. shRNA knockdown experiments demonstrated that the viability of primary AML cells, but not normal CD34+ cells, depended on *FOXM1* expression.

**Conclusions:**

We found that chemorefractory blasts from leukemias with varied genetic backgrounds expressed a common transcriptional program. In contrast to the notion that LSC quiescence confers resistance to chemotherapy we find that refractory blasts are both actively proliferating and enriched with LSC maintenance genes. Using primary patient material from a relevant clinical context we also provide further support for the role of FOXM1 in chemotherapy resistance, proliferation and stem cell function in AML.

**Supplementary Information:**

The online version contains supplementary material available at 10.1186/s12885-021-08839-9.

## Background

The greatest challenge in the management of acute myeloid leukemia (AML) is disease relapse which is due to chemoresistance of leukemia cells with disease repopulating potential. Previous studies have sought to identify in vivo resistance mechanisms by comparing blasts from presentation and relapse [1, 2]. However, most cases of relapsed AML remain chemoresponsive [3], indicating continued blast cell chemosensitivity, even though they are derived from an upstream chemoresistant leukemic stem or progenitor cell. Indeed, the genetic and transcriptional features of surviving leukemia cells shortly after completion of induction chemotherapy have yet to be defined. One prevalent hypothesis is that quiescence enables leukemic stem and progenitor cells (LSCs) to evade chemotherapy [4]. However, the evidence that LSCs are indeed quiescent is indirect and contradictory. In certain leukemia models, the LSC compartment may be both large and actively proliferating [5, 6]. Related to this, it is unclear how quiescence could account for primary refractory disease where a significant proportion of blasts survive and proliferate.

To identify features characteristic of chemoresistant AML cells we developed a protocol to study primary refractory disease, thereby facilitating comparison in the same patient of the transcriptome and mutational status of AML blast cells at presentation with those recovered immediately upon induction failure. In particular, we found that chemorefractory blasts from leukemias with varied genetic backgrounds expressed a common transcriptional program, signifying that these cells were more proliferative and less well differentiated. In contrast to the notion that LSC quiescence confers resistance to chemotherapy, we found that refractory blasts are both actively proliferating and enriched for expression of a LSC maintenance gene signature. In addition, we found that chemorefractory AML blast cells exhibit higher expression of genes bound by the Forkhead factor FOXM1. FOXM1 expression predicts for adverse outcome in a range of cancers [7, 8] and is highly expressed in AML where it is required for proliferation, in keeping with its binding to cell cycle gene homology regions (CHR) via its interaction with the MuvB complex [9–11]. Using primary patient material we provide further support for an important role for FOXM1 in AML.

## Methods

### Primary AML samples and cell lines

Primary human AML and normal mobilised peripheral blood samples were from the Manchester Cancer Research Centre Tissue Biobank (approved by the South Manchester Research Ethics Committee). Their use was authorized by the Tissue Biobank’s scientific sub-committee, with the informed consent of donors. THP1, K562, HL60 and OCI-AML3 cells were acquired from DMSZ (Braunschweig, Germany).

### Cell culture and colony-forming cell assays

Cell lines were cultured in RPMI 1640 with 10% fetal bovine serum (FBS; Sigma Aldrich, St. Louis, MO) and 50 U/ml penicillin-streptomycin (Thermo Fisher Scientific, Waltham, MA). Primary AML samples used for *FOXM1* knockdown experiments were expanded on a monolayer of murine MS-5 stromal cells prior to cryopreservation. Cells were thawed 7 days prior to transduction, recovered on MS-5 monolayers, then cultured in α-MEM medium (Thermo Fisher Scientific) supplemented with 12.5% heat-inactivated FBS, 12.5% heat-inactivated horse serum, 2 mM L-glutamine, 57.2 μM β-mercaptoethanol, 1 μM hydrocortisone (Sigma Aldrich) and IL-3, G-CSF and TPO (all at 20 ng/ml; Peprotech, Rocky Hill, NJ). Normal CD34^+^ cells were cultured in α-MEM medium (Thermo Fisher Scientific) supplemented with 12.5% heat-inactivated FBS, 12.5% heat-inactivated horse serum, 2 mM L-glutamine, 57.2 μM β-mercaptoethanol, 1 μM hydrocortisone (Sigma Aldrich), 1 μM StemRegenin 1 (Stem Cell Technologies, Vancouver, Canada) and SCF, FLT3L and TPO (all at 50 ng/ml; Peprotech).

Colony-forming cell (CFC) assays were performed at a density of 10^7^/ml in human methylcellulose medium (H4320, Stem Cell Technologies) with 3 μg/mL puromycin (Sigma Aldrich) and the appropriate combination of cytokines (as detailed above). Proliferation assays were performed at a cell density of 25 × 10^3^/ml in the appropriate medium with 3 μg/ml puromycin. Live cells were enumerated by analysing 100 μl of cell suspension using an Attune™ NxT Flow Cytometer (Thermo Fisher Scientific).

### Flow cytometry

**Fig. 1:**
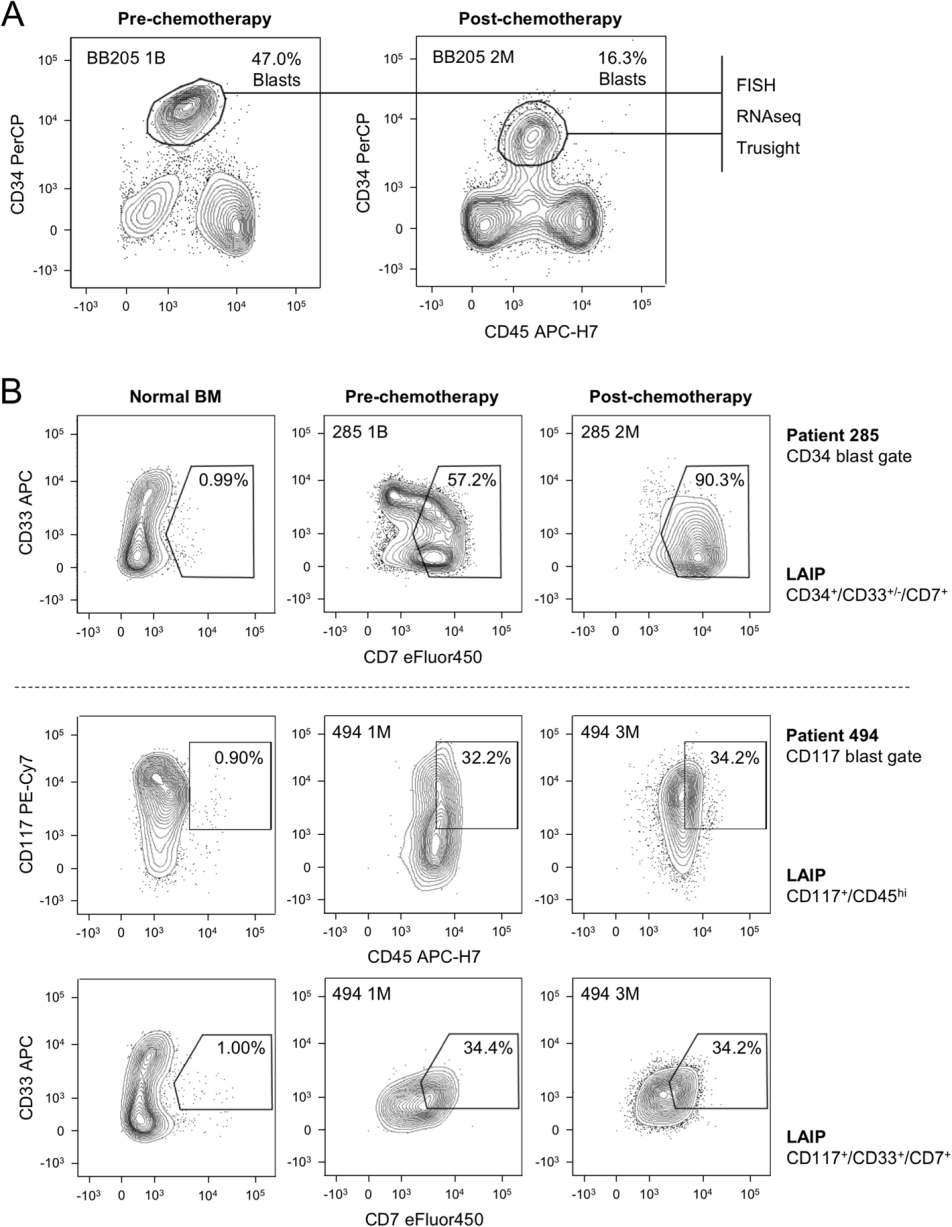
**A** Representative flow cytometry scatter plots show gating strategy for leukemic blast cell sorting. BB numbers indicate Biobank identifier. **B** Flow cytometry scatter plots showing the highest frequency leukemia associated immunophenotypes (LAIPs) identified in the indicated primary AML samples using a difference-from-normal gating strategy

Flow cytometry was performed using an LSR II flow cytometer (BD Biosciences, Franklin Lakes, NJ). FlowJo v10.6.1 (BD Biosciences) was used to analyze data. The antibody panel (Table S1) was adapted from Freeman et al. [12] to allow blast gating and identification of leukemia associated immunophenotypes (LAIPs; Figs. 1A-B, S1A-B and S2A). CD3 was added to Tube 1 for isolation of T cells to provide germ line DNA for targeted sequencing. Blasts and T-cells were flow sorted using a FACSAria™ III (BD Biosciences). CD38 expression cut-offs for identifying LSCs and progenitors were defined using the upper limit of the red cell fraction (CD38^low^) and the median of the red cell fraction (CD38^−^).

For cell cycle analysis sorted leukemic blasts or drug-treated primary AML cells were suspended in ice-cold 70% methanol in H_2_O and fixed at − 20 °C overnight. Cells were then washed twice in PBS, suspended in propidium iodide staining solution (20 μg/ml Propidium Iodide (Sigma Aldrich) and 500 μg/ml RNAse (Sigma Aldrich) in PBS) and incubated for 30 min at 37 °C. Samples were then analysed using a BD LSRII flow cytometer (BD Biosciences). An Annexin V Apoptosis Detection Kit (Thermo Fisher Scientific) was used to stain transduced cells 4 days after puromycin selection according to the manufacturer’s protocol. Samples were analysed using a BD LSRII flow cytometer (BD Biosciences).

### Cell viability assays

5 × 10^3^ cells were plated in each well of a 96-well plate with media containing a serial dilution of RCM-1 (Cambridge Bioscience) or Thiostrepton (Cambridge Bioscience). Plates were incubated for 96 h at 37 °C. 20 μl of 140 μg/mL resazurin (Sigma Aldrich) was added to each well. Plates were then incubated for a further 4 h and read using a POLARstar Omega plate reader (BMG Labtech, Aylesbury, UK).

### Fluorescence in situ hybridization (FISH)

Where numbers permitted, ~ 10,000 sorted blasts were fixed in methanol:acetic acid (3:1), slide mounted and subjected to FISH. Monosomy 7 provided a target in 3/6 leukemias and was detected using a Vysis D7S522/CEP7 FISH probe kit (Abbott Molecular, Abbott Park, IL).

### Targeted DNA sequencing with TruSight myeloid panel

DNA was extracted from sorted blast and T-cell populations using a QIAamp DNA Micro Kit (Qiagen, Manchester, UK). Where cell numbers were < 5 × 10^4^ DNA was amplified using a REPLI-g mini kit (Qiagen). Genomic DNA was then subjected to targeted next generation sequencing (NGS) using a NextSeq 500 sequencer (Illumina, San Diego, CA). The TruSight Myeloid Sequencing Panel (Illumina) targets 54 genes recurrently mutated in myeloid neoplasms using a proprietary multiplexed oligonucleotide pool covering each region of interest. Libraries were generated according to the manufacturer’s protocol. Briefly, genomic DNA was quantified using a Qubit DNA BR assay kit (Life Technologies, Carlsbad, CA) and diluted to 50 ng in 96 well plates. Oligonucleotides were hybridized to regions of interest, followed by an extension-ligation reaction and PCR amplification, incorporating unique combinations of i5/i7 index sequences to permit multiplexing up to 96 samples per sequencing run. Successful amplification was confirmed using a DNA 1000 kit and the 2100 Bioanalyzer system (Agilent Technologies, CA). Libraries were purified using AMPure magnetic beads (Agencourt, Brea, CA) and bead-normalized according to the TruSight protocol. Libraries were pooled (5 μL per library, 96 per pool) and quantified by PCR to determine molarity for loading onto the NextSeq flow cell. Paired end (150 bp) sequencing was performed on the NextSeq 500 sequencer (Illumina) with 96 samples multiplexed on a single NextSeq 500 High Output run (300 cycles).

Data analysis was performed within Illumina’s online BaseSpace genomics analysis platform. FASTQ files were aligned to human genome reference GRCh37/hg19 by the TruSeq Amplicon App (v2.0; Illumina) using a banded Smith-Waterman algorithm. Variant calling was performed by Somatic Variant Caller (v4.0.13.1; Illumina) using default parameters. The resulting gVCF files were uploaded to Variant Studio (v2.2.3; Illumina) for downstream filtering and annotation (from RefSeq database) of high confidence variants. Variant allele frequency was calculated as the fraction of mutated reads versus total number of reads covering that base. Mutations were considered present if they had a variant allele frequency > 10%, a total depth > 500, were absent from germline (T-cells) and were annotated in the COSMIC database [13].

### RNA extraction, sequencing and data analysis

Total RNA was extracted from 10^3^ to 10^5^ sorted blasts using QIAshredder spin columns and a RNeasy® Plus Micro kit (Qiagen). Prior to sequencing RNA integrity was checked using a 2100 Bioanalyzer (Agilent Technologies, Santa Clara, CA). Total RNA yield from the sorted populations ranged from 3.5-475 ng. To ensure consistency the sample with the lowest yield was used to define the input (3.5 ng) for amplification of all samples. Amplification was performed using a SMARTer Stranded Total RNA-Seq Pico input kit (Clontech, Mountain View, CA). Sequencing was performed using a NextSeq desktop sequencing system (Illumina). A single run (400 M reads) of 151 bp paired-end sequencing produced a mean of 31.4 M reads per sample (range 29.3–33.5 M). Reads were aligned to the human genome (hg38) using STAR v2.4.2a [14]. DEseq2 was used to calculate FPKM (fragments per kilobase of transcript per million mapped reads) values for each transcript [15]. Principal component analysis was performed using ggplot2 [16] and heatmaps were generated using R. Gene ontology analysis was performed using the DAVID Bioinformatics Resource 6.7 [17]. Gene set enrichment analyses were performed using GSEA v4.0.1 software [18]. Expressed protein coding genes were ranked using a signal-to-noise metric. Potential transcriptional regulators were identified by screening genes upregulated in post-chemotherapy blasts with consensus transcription factor targets using Enrichr [19, 20]. FOXM1 transcriptional targets were from ChIPseq experiments performed by Chen et al. [21].

### Quantitative PCR

RNA was extracted from primary AML, normal CD34+ cells and cell lines using an RNeasy® Plus Micro kit (Qiagen). cDNA was generated using a High Capacity cDNA Reverse Transcription Kit (Thermo Fisher Scientific). qPCR reactions were performed in MicroAmp® optical 384-well reaction plates and analysed using a QuantStudio® 5 PCR system (Applied Biosystems). Reactions were performed in triplicate and included primers for β-Actin (*ACTB*) as a housekeeping gene. Primers were designed using the Universal Probe Library (UPL) Assay Design Center (Roche, Basel, Switzerland) and purchased from Integrated DNA Technologies (IDT, Coralville, IA). Raw fluorescence data was converted to Ct values using the Thermo Fisher Cloud facility (Waltham, MA) and normalised to *ACTB*. Primers were (i) *ACTB* (F) ATTGGCAATGAGCGGTTC, (R) GGATGCCACAGGACTCCAT, UPL probe #11 and (ii) *FOXM1* (F) AGAAACGGGAGACCTGTGC, (R) CCACTGGATGTTGGATAGGC, UPL probe #74.

### CD34 enrichment

CD34^+^ cells from healthy donor mobilised peripheral blood were enriched by magnetic-activated cell sorting (MACS). Briefly, cryopreserved samples were thawed and dead cells removed with a Dead Cell Removal Kit (Miltenyi Biotec, Bergisch Gladbach, Germany). Double CD34^+^ enrichment was then performed using UltraPure CD34 MicroBeads (Miltenyi Biotec), according to the manufacturer’s protocol, resulting in CD34^+^ cell purity > 95%. Cells were maintained in culture for 5 days prior to transduction to allow cell recovery.

### Lentiviral vector construction and transduction

pLKO.1-puro (Sigma Aldrich) was used to express short hairpin RNAs (shRNAs) targeting human *FOXM1* (*FOXM1* KD#1: 5′-GCCCAACAGGAGUCUAAUCAA-3′; *FOXM1* KD#2: 5′-GCCAAUCGUUCUCUGACAGAA-3′). Oligonucleotides (IDT) were annealed through incubation at 98 °C for 5 min, and cooling to room temperature. The vector was digested with AgeI and EcoRI (New England Biolabs, Ipswich, MA) and ligated to the annealed oligonucleotides. Cloning was confirmed using Sanger sequencing. A pLKO.1-puro vector containing a non-targeting oligonucleotide (SHC002) was used as a control. Primer sequences were as follows:

- *FOXM1 KD#1*
- (F) CCGGGCCCAACAGGAGTCTAATCAACTCGAGTTGATTAGACTCCTGTTGGGCTTTTTG
- (R) AATTCAAAAAGCCCAACAGGAGTCTAATCAACTCGAGTTGATTAGACTCCTGTTGGGC
- *FOXM1 KD#2*
- (F) CCGGGCCAATCGTTCTCTGACAGAACTCGAGTTCTGTCAGAGAACGATTGGCTTTTTG
- (R) AATTCAAAAAGCCAATCGTTCTCTGACAGAACTCGAGTTCTGTCAGAGAACGATTGGC

Lentiviral particles were generated through polyethylenimine-mediated transfection of HEK293T cells. AML or normal human CD34^+^ cells were twice cultured overnight in fresh viral supernatant supplemented with the relevant cytokines (as above), polybrene (8 μg/mL; Merck Group, Darmstadt, Germany), and DEAE-Dextran (4 μg/mL; Sigma Aldrich). Cells were subsequently transferred into fresh media (as above) with 3 μg/ml puromycin and cultured for 48 h prior to analysis. Cells were maintained in puromycin in all subsequent assays.

## Results

To characterise the genetic and transcriptional features of chemorefractory primary AML samples, we performed molecular genetic and RNA sequencing analyses of flow sorted, paired blast samples collected from six patients at presentation and following one (*n* = 5) or two (*n* = 1) courses of induction chemotherapy (Table 1). Blasts were flow sorted according to either a CD45^low/int^CD34^+^ (*n* = 5) or, in a case which lacked CD34 expression, CD45^low/int^CD117^+^ (*n* = 1) immunophenotype (Fig. 1A and S1A-B). The leukemic origin of sorted blast populations was confirmed using fluorescence in situ hybridization (FISH) where suitable chromosomal markers permitted (Table 2) and targeted sequencing of 54 genes recurrently mutated in myeloid neoplasms (Tables 3 and S2). Over and above flow sorting based on the above mentioned immunophenotypes, samples were also analysed using an extended flow panel to identify leukemia-associated immunophenotypes (LAIPs; Figs. 1B, S2A and Table 3) to provide additional confirmation that post-chemotherapy cells were leukemic and not regenerating normal progenitors [12]. In keeping with the concept that different genetic sub-clones exhibit different levels of chemosensitivity, molecular genetic analysis revealed the presence of early clonal selection following the first cycle of chemotherapy, with the emergence of new subclones (patients 64, 285, and 349), the loss of pre-treatment subclones (patients 285 and 494) or a reduction in size of the dominant clone (patients 64 and 285; Tables 3 and S2). The identified LAIPs were observed both before and after chemotherapy in each case, although the change in percentage of BM cells positive for each LAIP did vary widely (Table 3, Figs. 1B and S2A). There was no consistent or statistically significant change in expression of cell surface markers between pre- and post-chemotherapy samples (Fig. S2B). Thus, even in patients proving to be refractory to their first cycle of chemotherapy there was nevertheless often evidence of chemotherapy-induced clonal selection.

**Table 1:** Clinical characteristics of primary AML patient samples used for RNA sequencing

	Pre-chemotherapy							Post-chemotherapy				
BB^a^	Age range	Sex	WHO (2016)	WCC	Karyotype^b^	Sample	Induction^c^	Response	Sample	Blast %^e^	Days since start of Rx	Outcome
64	40–59	M	AML with inv.(3)	14.1	Inv(3)	PB	ADE + GO	Borderline refractory with persistent cytogenetic abnormality	BM	5%	33	Relapsed and died following HSCT^f^
121	16–39	F	AML with inv.(3)	6.5	Inv(3)	BM	DA + GO	Refractory	BM	30%	20	Transient remission with salvage therapy; then relapsed and died
205	40–59	F	Therapy-related myeloid neoplasm (t-AML)	12.6	Monosomal	PB	DA (90) FLAG-IDA^4^	Refractory	BM	12%	62^d^	Relapsed and died following HSCT^f^
285	60–79	M	AML with myelodysplasia-related changes	3.9	Normal	PB	DA	Refractory	BM	30%	32	Induction failure; died
349	40–59	M	AML, not otherwise specified	2.4	Normal	BM	DA	Refractory	BM	70%	38	Transient remission with salvage therapy; then relapsed and died
494	60–79	M	AML, not otherwise specified	30.2	Monosomal	BM	DA	Refractory	BM	58%	32	Induction failure; died

^a^Biobank identifier

^b^See Table 2 for details

^c^Induction regimes: *ADE* Cytarabine, Daunorubicin, Etoposide; *GO* Gemtuzumab ozogamycin; *DA* Daunorubicin (standard dose 60 mg/m^2^, patient 205 received 90 mg/m^2^ as part of a clinical trial), Cytarabine; *FLAG-IDA* Fludarabine, Cytarabine, G-CSF, Idarubicin

^d^The post treatment sample for patient 205 was collected after a second cycle of chemotherapy. All other post-treatment samples were collected following induction chemotherapy

^e^Blast percentage following treatment as determined by clinical service flow cytometry (FC). FC not performed for sample 64 2 M; results are shown for trephine histopathology (5%)

^f^HSCT performed in the presence of persistent cytogenetic abnormality but morphological remission

WCC, white cell count (× 10^9^/l); PB, peripheral blood; BM, bone marrow; HSCT, hematopoietic stem cell transplantation; Rx, treatment

**Table 2:** Fluorescence in situ hybridization (FISH) on sorted blasts

Biobank number	Cytogenetic details	FISH pre-chemotherapy	FISH post-chemotherapy	Probe used
64	46, XY, inv.(3)(q21q26), del(7)(q22) [10]	Failed	Insufficient material	NA
121	45, XX, inv.(3)(q21q26), −7[8]/46, XX [2]	44/45 1G1O 1/45 1G	83/120 1G1O 37/120 1G	Vysis D7S522/CEP7
205	44, XX, add(3)(p25), −5, −7[12]	61/100 1G1O 39/100 1G	Insufficient material	Vysis D7S522/CEP7
285	Normal			
349	Normal			
494	45 ~ 49, XY, −4, −5, −7, del(9)(q?22),? der(15;17) (q10;q10), + 21, del(22)(q13), + 2 ~ 5mar[cp10]	Insufficient material	92/100 1G1O 8/100 1G	Vysis D7S522/CEP7

Where sufficient cells were available, FISH was used to confirm clonality of sorted blast populations. Samples 285 and 349 had normal cytogenetics and no target for FISH. Samples 121, 205 and 494 had monosomy 7 which was detected using two probes: CEP7 (green probe targeting the centromere, 7p11.1-q11.1) and D7S522 (orange probe targeting 7q31). 1G1O, 1 green and 1 orange signal per cell; 1G, 1 green signal per cell

**Table 3:** Summary of mutation analyses of sorted blast populations

Clinical details			Molecular Genetics					Leukemia-associated immunophenotype (LAIP)		
**BB**^a^	**Response**	**Karyotype**	**Gene**	**Mutation**	**Amino acid change**	**Variant allele frequency**		**LAIP details**	**Pre-C %**	**Post-C %**
						**Pre-C %**	**Post-C %**			
64	Borderline refractory with persistent inv.(3)	Inv(3)	*KRAS* *GATA2* *PTEN* *NOTCH1*	Missense Missense Missense Missense	G13D L321V G251D D2108N	49 40 0 0	26 27 12 22	CD34^hi^/CD13^+^	10.4	24.4
121	Refractory	Inv(3)	*PTPN11*	Missense	G503A	45	50	CD34^+^/CD33^+/−^/HLADR^wk/−^CD34^hi^/CD13^+^	57.1 20.6	21.7 50.4
205	Refractory	Monosomal	*PTPN11* *DNMT3A*	Missense Missense	E76Q A192G	24 16	Failed	CD34^hi^/CD33^+^ CD34^+^/CD33^+^/HLADR^wk^	70.9 25.0	5.12 30.9
285	Refractory	Normal	*FLT3* *TET2* *KIT* *U2AF1* *EZH2* *TET2* *TP53* *WT1*	Missense Stop-gain Missense Missense Frameshift Missense Missense Missense	D835E R1452X P623L S34F N/A D1242G E307G R401K	47 41 11 0 0 0 0 0	31 35 0 26 41 20 16 12	CD34^+^/CD33^+/−^/CD7^+^	57.2	90.3
349	Refractory	Normal	*IDH2* *TET2*	Missense Stop-gain	R172K Q1539X	60 0	57 12	CD117^hi^	9.11	5.98
494	Refractory	Monosomal	*TP53* *JAK2* *FBXW7*	Missense Missense Missense	R234H N533D E471K	94 12 11	99 0 0	CD117^+^/CD45^hi^ CD117^+^/CD33^+^/CD7^+^	32.2 34.4	34.2 34.2

^a^Biobank identifier

Summary of Trusight Myeloid Sequencing Panel targeted next generation sequencing (NextSeq 500 System, Illumina) of sorted blast populations (see Table S2 for complete data). Also shown is the frequency of the indicated leukemia associated immunophenotype (LAIP) for each sample. C, chemotherapy

**Fig. 2:**
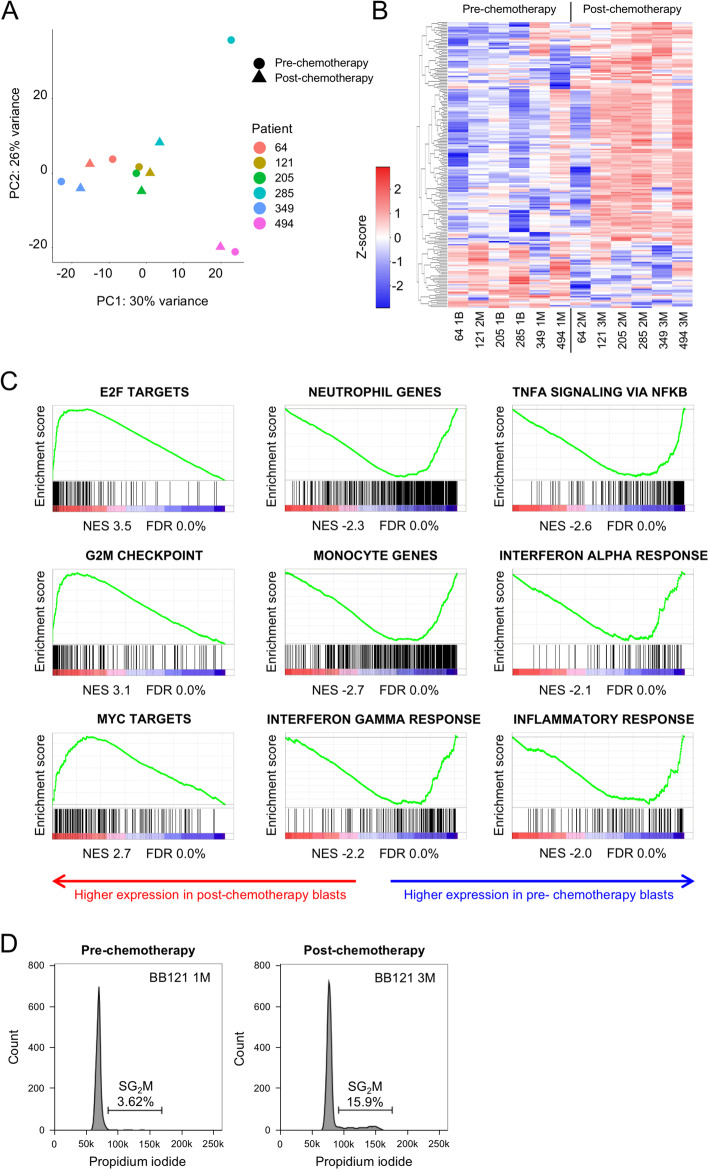
**A** Principal component analysis (PCA). **B** Heatmap shows differentially expressed genes (150 upregulated and 42 downregulated; paired t-test < 0.05, mean fold change > 1.5 or < 0.67). **C** Gene set enrichment analysis plots. NES, normalised enrichment score; FDR, false discovery rate. **D** Flow cytometry histograms show propidium iodide staining of flow sorted AML blasts pre- and post-chemotherapy from patient BB121

We next performed RNA sequencing of the paired pre- and post-chemotherapy CD45^low/int^CD34^+^ or CD45^low/int^CD117^+^ sorted blast cell populations. As expected, given the genetic heterogeneity of the analysed samples, principal component analysis (PCA) of 7123 expressed protein-coding genes revealed significant transcriptional differences between the six leukemias (Fig. 2A; Table S3). By contrast, post-chemotherapy samples were generally quite similar to pre-chemotherapy samples from the same leukemia. The sole exception was the pair of samples from patient 285, the case with the most substantial chemotherapy-induced clonal evolution (Fig. 2A; Table 3). To search for consistent changes in the transcriptome of pre- and post-chemotherapy samples, we performed differential gene expression analysis. We identified 150 and 42 genes as significantly up or down regulated respectively (*p* < 0.05 by paired t-test, fold change > 1.5 or < 0.67; Fig. 2B and Table S4). Among significantly up regulated genes there was enrichment for Gene Ontology Biological Process terms “cell division” (GO:0051301; *P* = 10^− 21^) and “mitotic nuclear division” (GO:0007067; *P* = 10^− 13^). There was no significant enrichment for any term among significantly down regulated genes [17]. To characterise the transcriptional changes in post-chemotherapy versus pre-chemotherapy AML blasts in greater depth and to detect co-ordinate changes in expression of a priori defined gene sets, we also performed pre-ranked Gene Set Enrichment Analysis (GSEA) using a signal-to-noise ranking metric [18]. Evaluation of the pattern of expression of the Molecular Signatures Database Hallmark Gene Set collection, each of which conveys a specific biological state or process and displays coherent expression [22], demonstrated strong up regulation of “E2F target” genes, “G2M cell cycle checkpoint” genes and “MYC target” genes in post- versus pre-chemotherapy AML blast cells (Fig. 2C). In addition, there was down regulation of “inflammatory response” genes, and genes regulating “TNFA signalling via NFKB”, “Interferon gamma response” and “Interferon alpha response” (Fig. 2C). Given that these latter gene sets are characteristic of terminally differentiated myeloid cells we performed similar GSEA analyses using sets of genes preferentially expressed in normal human monocytes and neutrophils (Table S4) [23, 24] and observed that these gene sets were also strongly down regulated in post- versus pre-chemotherapy AML blast cells (Fig. 2C). Together these data indicate that AML blast cells surviving chemotherapy are more proliferative and less well differentiated than those at presentation. One sample from patient 121 had sufficient residual sorted blasts before and after chemotherapy to permit cell cycle analysis: propidium iodide staining confirmed a remarkable 4-fold higher percentage of blast cells in SG2M in post- versus pre-chemotherapy AML blast cells (Fig. 2D).

**Fig. 3:**
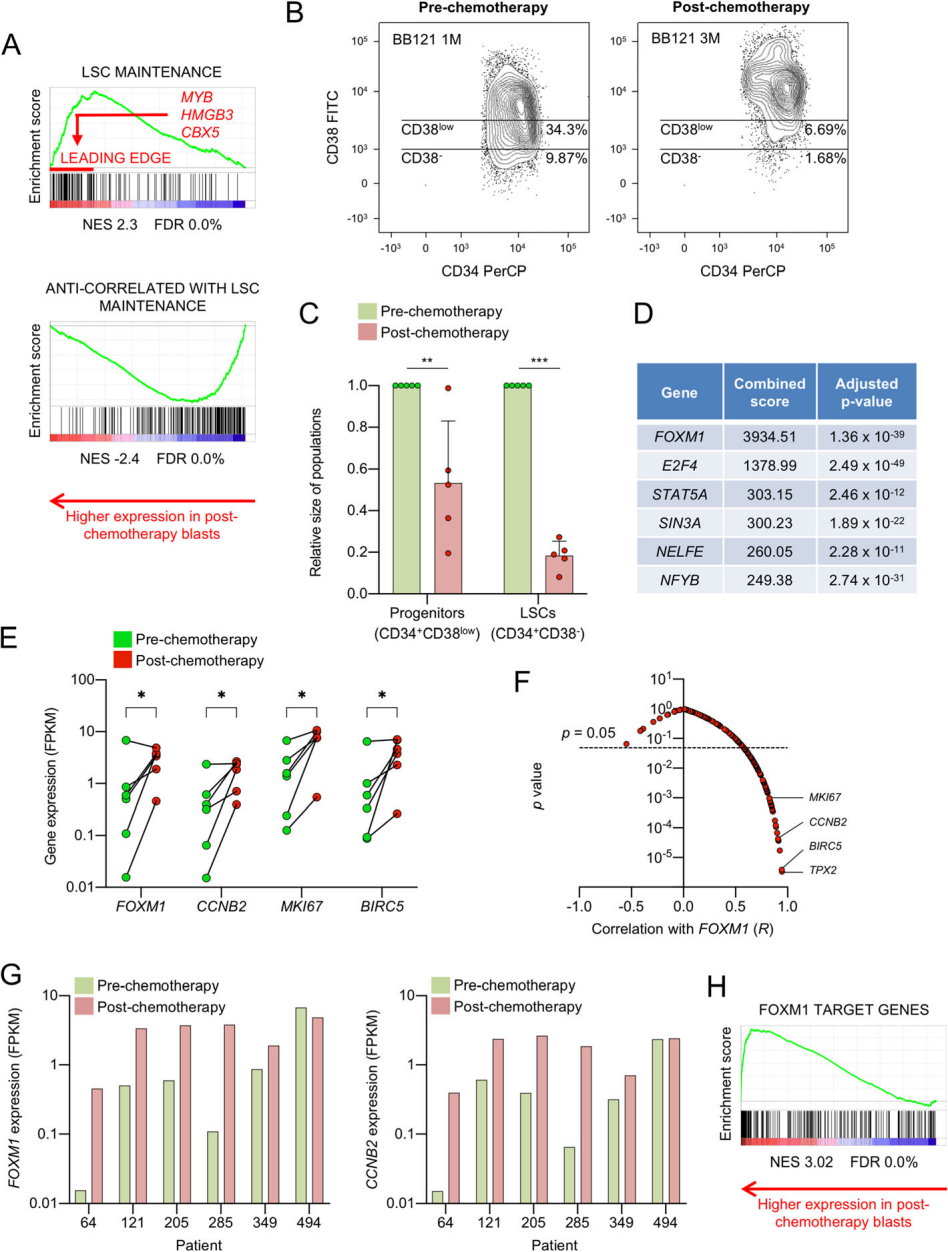
**A** Gene set enrichment analysis (GSEA) plots. NES, normalised enrichment score; FDR, false discovery rate. **B** Representative flow cytometry scatter plots show the relative size of the immunophenotypic leukemia stem and progenitor cell populations pre- and post-chemotherapy. **C** Mean ± SD relative size of AML stem and progenitor populations (*n* = 5). Sample 494 was excluded as the blasts lacked CD34 expression at presentation. ** *P* < 0.01, *** P < 0.001 by unpaired t-test. BB numbers indicate Biobank identifier. **D** Table shows enrichment of gene sets directly bound by the indicated transcription factors among the 150 genes upregulated in post-chemotherapy AML blasts. The combined score is the product of the logarithm of the adjusted *p*-value and the z-score (20). The analysis was performed using Enrichr (19). **E** Expression of the indicated genes in pre- and post-chemotherapy flow sorted AML blast populations (*n* = 6). FPKM, fragments per kilobase of transcript per million mapped reads. * *P* < 0.05 by ratio paired t-test. **F** Scatter plot shows correlations between absolute expression values (FPKM) for *FOXM1* and the 150 genes significantly upregulated in post-chemotherapy blasts (Table S5; *n* = 12). *R* represents the Pearson product moment correlation coefficient. **G** Bar charts show expression of the indicated genes in all samples. FPKM, fragments per kilobase of transcript per million mapped reads. **H** GSEA plot shows significantly enriched expression of FOXM1 target genes in post- versus pre-chemotherapy AML blasts. NES, normalised enrichment score; FDR, false discovery rate

While we observed these transcriptional features in primary human chemorefractory AML cases, they are nevertheless reminiscent of our prior findings in a murine model of human *MLL*-translocated AML where we found the LSC compartment to be more proliferative and less well differentiated than downstream cells [6, 25]. Indeed cross-species comparison of the gene set associated with LSC maintenance in murine MLL-AF9 AML cells showed significantly higher expression in post- versus pre-chemotherapy human AML cells, and vice versa for genes whose expression is anti-correlated with leukemia stem cell activity (Table S4 and Fig. 3A). Intriguingly, leading edge analysis of the genes driving enriched expression of the LSC maintenance signature in post- versus pre-chemotherapy blast cells revealed the presence of *MYB*, *HMGB3* (High Mobility Group Box 3) and *CBX5* (Chromobox 5) the three genes which, when co-expressed, suffice for Hox/Meis-independent immortalization of myeloid progenitor cells (Fig. 3A) [6].

Our analysis of LAIPs uncovered an additional unexpected finding: a consistent reduction in the percentage of immunophenotypic LSCs (CD34^+^CD38^−^) following chemotherapy. Consistent with reports that LSC frequencies at presentation predict for poor survival [26], the frequency of immunophenotypic LSCs was typically high at presentation (median 9.9%, range 0.36–47.3%; *n* = 5). Following chemotherapy, among surviving AML cells, there was a significant reduction in the proportion of cells with either an LSC immunophenotype (CD34^+^CD38^−^) or a progenitor immunophenotype (Figs. 3B-C). The seeming disconnect between a relative increase in expression of genes associated with LSC maintenance and a reduction in numbers of immunophenotypically-defined LSCs is striking but may be explained by the imperfect ability of cell surface markers such as CD34 and CD38 to define functional LSC potential. Related to this, there is plentiful evidence that LSCs may be aberrantly self-renewing downstream progenitor cells expressing cell surface markers more characteristic of mature myeloid populations [27].

Next, to identify transcription factor regulators of the refractory blast gene expression signature, which may serve as candidate therapeutic targets, we performed an in silico analysis using Enrichr [7, 28], a bioinformatics resource which facilitates analysis of experimentally derived gene sets. Among the 150 genes upregulated in refractory AML blast cells (Table S4), the most significant enrichment among the 92 sets of transcription factor binding sites interrogated was for genes bound by the Forkhead transcription factor FOXM1 (Fig. 3D), whose expression is known to predict for adverse outcomes in cancer [7, 8]. Thus, among genes up regulated in post- versus pre-chemotherapy AML blasts there is significant enrichment for those known to be direct targets of FOXM1 binding. Consistent with this, expression of *FOXM1* increased markedly following chemotherapy in five of six cases (Fig. 3E).

To further assess the contribution of FOXM1 to the refractory blast gene signature we performed correlation analyses between the absolute expression values (FPKM) of *FOXM1* and the genes significantly upregulated in refractory blasts (Table S5). We hypothesised that genes directly controlled by FOXM1 were likely to exhibit highly correlated gene expression patterns across all 12 pre- and post-chemotherapy samples tested. 82/150 genes were significantly, positively correlated with *FOXM1* expression (Pearson *R* > 0.5, *p* < 0.05; Fig. 3F). In keeping with the known binding of FOXM1 to DNA sequences termed cell cycle gene homology regions (CHR) via its interaction with the MuvB complex [9] several of the most highly correlated genes were regulators or markers of cell cycle progression (*CCNB2, MKI67*, *TPX2 & BIRC5*), suggesting that FOXM1 may serve to sustain post-chemotherapy AML cell proliferation (Figs. 3E-F and S3A). Of note, the one leukemia that did not exhibit increased *FOXM1* expression following chemotherapy (494) had the highest baseline expression of refractory-associated genes, including *FOXM1* and associated cell cycle genes (Figs. 2B, Fig. 3G and S3B). Patient 494 also had the highest blast count at presentation and the most aggressive clinical course (remaining refractory to salvage chemotherapy), indicating highly aggressive, proliferative disease (Table 1).

Having identified abundant FOXM1 targets among the 150 genes upregulated in refractory blasts we next sought to assess the expression of all FOXM1 targets in pre- versus post-chemotherapy blasts. We made use of a ChIP sequencing dataset generated from the human osteosarcoma cell line U2OS [21]. In published datasets, FOXM1 predominantly binds the 5’UTR and promoter regions of target genes [29]. Of the 270 high confidence FOXM1 targets in U2OS cells, 206 occupied positions ±1kB from a transcription start site. In GSEA, there was substantial and highly significant enrichment of genes bound at the promoter by FOXM1 among genes up regulated in post-chemotherapy AML blasts (Table S4 and Fig. 3H). Of note, there was minimal overlap between the FOXM1 target and LSC maintenance gene sets, with only *PTTG1* (Pituitary Tumor-Transforming Gene 1), *ZADH2* (Zinc Binding Alcohol Dehydrogenase Domain Containing 2) and *DTYMK* (Deoxythymidylate Kinase) found in both sets. This is in keeping with our prior studies which demonstrated that the LSC maintenance signature reflects a leukemic self-renewal program predominantly driven by expression of *MYB* [6], rather than merely a signature of cell proliferation.

**Fig. 4:**
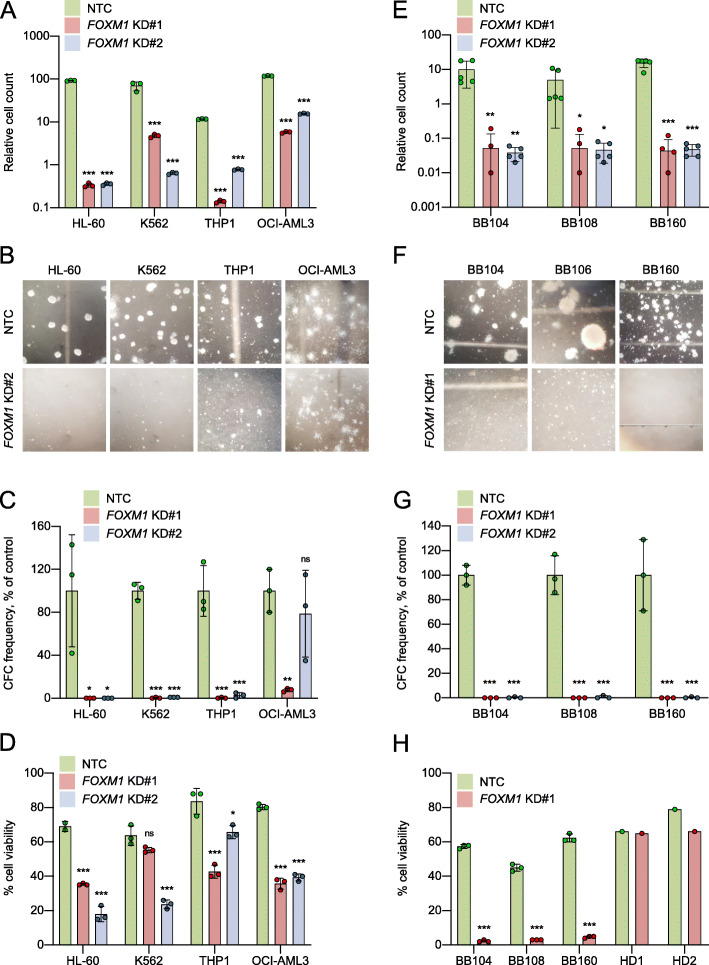
**A**-**D** AML cell lines were infected with lentivirus targeting FOXM1 for knockdown (KD#1 and KD#2) or a non-targeting control (NTC). Cells were plated into proliferation or colony-forming cell (CFC) assays after 48 h of puromycin drug selection (i.e. Day 0). Apoptosis assays were performed after four days of puromycin drug selection. **A** *Bar chart shows mean ± SD (n = 3) cell count on Day 7 of culture in the indicated conditions. Cell counts are shown relative to Day 0.* **B** Representative images of CFC assays. Bar charts show *mean ± SD* (*n* = 3) (**C**) CFC frequencies or (**D**) cell viability in the indicated conditions *for the indicated cell lines*. **E**-**H** Primary AML cells (BB104, BB108 & BB160) and normal CD34^+^ HSPCs from healthy donors (HD1 & HD2, apoptosis assays only) were infected with lentivirus targeting FOXM1 for knockdown (KD#1 and KD#2) or a non-targeting control (NTC). Cells were plated into proliferation or colony-forming cell (CFC) assays after 48 h of puromycin drug selection (i.e. Day 0). Apoptosis assays were performed after four days of puromycin drug selection. **E** *Bar chart shows mean ± SD (n = 3) cell count on Day 7 of culture in the indicated conditions. Cell counts are shown relative to Day 0.* **F** Representative images of CFC assays. Bar charts show *mean ± SD (n = 3) (****G****)* CFC frequencies or (H) cell viability in the indicated conditions *for the indicated cells*. * *P* < 0.05, ** *P* < 0.01, *** *P* < 0.001 by one-way ANOVA *with Dunnett’s multiple comparison test (****A****-****G****)* or unpaired t-test (**H**). *BB numbers indicate Biobank identifier*

FOXM1 is more highly expressed in primary human AML cells than in normal hematopoietic stem and progenitor cells (HSPCs) and is required for their proliferation [10, 11] and its expression has been linked to chemoresistance in AML cell lines [30]. Levels of nuclear FOXM1 have been correlated with patient treatment response [11], and in murine models of MLL-AF9 induced AML it is required for optimal LSC function [31]. To confirm and extend these findings, we studied the effect of shRNA-induced knockdown in four *FOXM1*-expressing human AML cell lines (Fig. S4A-B). *FOXM1* knockdown significantly reduced the capacity of all cell lines to proliferate in liquid culture and to form colonies in semi-solid medium (Figs. 4A-C and S4C). Interestingly, this effect was least pronounced in the NPM1 mutant cell line (OCI-AML3), consistent with previous studies suggesting that nuclear export of FOXM1 by mutant NPM1 contributes to the relative chemosensitivity of this leukemia [30]. To determine whether these findings were due to reduced cell proliferation or impaired viability we performed cell cycle analysis and Annexin-V apoptosis assays. Consistent changes in cell cycle status were not observed following *FOXM1* knockdown (Fig. S4D), whereas cell viability decreased significantly in all cell lines (Figs. 4D and S4E-F). To expand these findings, we performed shRNA-induced *FOXM1* knockdown in three cases of primary AML with an *MLL* gene rearrangement which all exhibited high *FOXM1* expression (Table S6, Figs. S4A and S5A). *FOXM1* knockdown prevented proliferation in liquid culture and abolished colony formation in all primary cells (Figs. 4E-G and S5B). The reduction in cell viability was even more pronounced than that observed in AML cell lines (Figs. 4H and S5C). To assess the potential of FOXM1 as a therapeutic target in AML we determined the effect of *FOXM1* knockdown on the viability of normal CD34^+^ HSPCs from two healthy donors (Figs. S4A and S5A). Consistent with recent reports [31], *FOXM1* knockdown did not induce apoptosis in normal HSPCs (Figs. 4H and S5C).

The compounds Thiostrepton and RCM-1 have been reported to inhibit FOXM1 activity [32, 33]. In contrast to *FOXM1* knockdown, following drug treatment we observed reduced cell growth due to apoptosis in both AML cells and in normal CD34^+^ HSPCs at similar IC_50_s (Figs. S6A-D). This is likely due to off target effects. For example, while thiostrepton binds FOXM1 [33] it also binds the proteasome [34] and the large subunit of the mitochondrial ribosome [35]. Likewise the mechanism by which RCM-1 promotes ubiquitination and proteasomal degradation of FOXM1 is unknown [32] and it is not clear what other proteins are concomitantly targeted for degradation.

All together these data confirm that by comparison with normal hematopoietic stem and progenitor cells, FOXM1 appears selectively required for leukemia cell proliferation.

## Discussion

Transcriptome studies in AML over the last two decades have been helpful in identifying critical genes and cellular pathways that contribute to leukemogenesis. However, the practical challenges of collecting post-chemotherapy material containing sufficient leukemic blasts for analysis have resulted in a lack of studies comparing paired pre- and post-chemotherapy samples. Post-chemotherapy bone marrow is often hypocellular and the majority of patients remit after induction chemotherapy. Our prospective biobanking strategy of collecting bone marrow from all patients at presentation and after induction has enabled accumulation of a set of paired pre- and post-chemotherapy samples from patients with refractory disease. These samples enabled us to perform detailed genetic and transcriptional characterisation of the blasts that survived chemotherapy.

Despite significant genetic heterogeneity we found that chemorefractory primary AML blasts shared common transcriptional features. Most striking was the enrichment of genes involved in cell cycle progression, implying that chemoresistant blasts were more proliferative than their chemosensitive counterparts. This was a remarkable finding given the prevalent hypothesis that AML cells survive chemotherapy because of replicative quiescence. While we cannot exclude the possibility that blasts in G0 survive chemotherapy then enter the cell cycle rapidly following completion of chemotherapy, this seems unlikely. If quiescence were a dominant mechanism of resistance but chemotherapy exposure was sufficient to stimulate widespread cell cycle entry we would expect to see improved outcomes following extended treatment courses or pulsed maintenance strategies. Whilst these approaches are effective in acute lymphoblastic leukemia, where the evidence for LSC quiescence is more compelling, they have not been effective in AML [36, 37]. Contrary to previous studies, our data argue against quiescence as a mechanism of chemoresistance, but are concordant with the clinical literature identifying high presentation blast count as a predictor of treatment failure [5, 38]. Our data are also consistent with murine studies where LSCs have been found to be both actively cycling, frequent and chemoresistant [5, 6].

TIM3 and CD47 are LSC markers that have generated considerable interest as potential targets of cellular or antibody-based immunotherapy [39, 40]. However, we found that the proportion of immunophenotypically defined LSCs fell following chemotherapy whilst the expression of stem cell genes increased. Previous studies have described a similar decoupling of stemness from immunophenotype in murine models of AML [41]. These findings have implications for the therapeutic targeting of LSCs based on cell surface immunophenotype, at least if treatment is delivered concomitantly with chemotherapy. Additional studies are required to confirm that the expression of potential targets is stable following chemotherapy exposure. If the cells that actually survive chemotherapy do not necessarily express putative LSC markers then their targeting is unlikely to improve patient outcomes.

Our data identifies the Forkhead transcription factor FOXM1 as both a candidate regulator of the chemorefractory gene expression program and also as essential for AML but not normal HSPC viability, at least in vitro. FOXM1 has been implicated in all major hallmarks of cancer and a major meta-analysis of expression signatures from ~ 18,000 human cancers identified the FOXM1 regulatory network as a major predictor of adverse outcome [8, 42]. Nuclear FOXM1 expression predicts treatment failure in intermediate risk AML and was found to drive proliferation and clonogenic potential in leukemic cell lines [10, 11]. Constitutive expression also conferred resistance to cytarabine in a murine myeloid leukemia model [11]. Our data, derived from primary patient material taken at clinically relevant time points, support these findings and provide an additional rationale for the therapeutic targeting of FOXM1.

A recent study by Sheng et al. demonstrated that FOXM1 was essential for LSC function in MLL-rearranged murine AML [31]. Whilst we found minimal overlap between FOXM1 target genes and the LSC maintenance programme, we cannot rule out indirect regulation. We also lack FOXM1 binding data from AML, which may differ from other cell types because FOXM1 is able to bind non-consensus sequences via co-factors [43]. Sheng et al. found that whilst FOXM1 promoted LSC quiescence in steady state conditions, conditional *FOXM1* knockout prevented leukemic repopulation of murine bone marrow following chemotherapy. These observations are concordant with our finding that the blasts repopulating human bone marrow after unsuccessful treatment express high levels of *FOXM1* and it targets. Importantly, our data suggest that the role of FOXM1 in AML is not limited to cases with MLL-rearrangement, but common to multiple genetic subtypes.

## Conclusions

Our work demonstrates that chemorefractory blasts from leukemias with varied genetic backgrounds nonetheless express a common transcriptional signature. Remarkably, we find that leukemic cells surviving chemotherapy are both enriched with LSC maintenance genes and are more proliferative and less well differentiated than those at presentation. These data suggest that replicative quiescence is not a dominant mechanism of resistance in AML. The refractory blast gene signature implicates the transcription factor FOXM1 and we provide further support for the role of FOXM1 in chemotherapy resistance, proliferation and stem cell function in AML.

## Supplementary Information

**Additional file 1: Table S1.** Antibody panel used for flow cytometry. **Table S6.** Primary AML samples used for *FOXM1* KD.

**Additional file 2: Table S2**. TruSight Myeloid Sequencing Panel results. **Table S3**. Expression values (FPKM) for expressed protein coding genes. **Table S4.** Gene sets. **Table S5**. Correlation of upregulated genes (Table S3) with *FOXM1* (FPKM).

**Additional file 3: Fig. S1.** Flow cytometry gating strategy. **Fig. S2.** Leukemia associated immunophenotypes (LAIPs). **Fig. S3.** Gene set enrichment analysis (GSEA) and *FOXM1* correlation analyses in post-chemotherapy blasts. **Fig. S4.** *FOXM1* KD in AML cell lines. **Fig. S5.** *FOXM1* knockdown in primary AML and normal CD34+ HSPCs. **Fig. S6.** FOXM1 inhibition in primary AML and normal CD34+ HSPCs.

## Data Availability

Raw data files are available at the Gene Expression Omnibus with accession number GSE162542 (https://www.ncbi.nlm.nih.gov/geo/query/acc.cgi?acc=GSE162542).

## Abbreviations

ACTB: Actin beta
AML: Acute myeloid leukemia
BIRC5: Baculoviral IAP repeat containing 5
BM: Bone marrow
CCNB2: Cyclin B2
cDNA: Complementary DNA
CFC: Colony forming cell
ChIP: Chromatin immunoprecipitation
FACS: Fluorescence-activated cell sorting
FBS: Foetal bovine serum
FISH: Fluorescence in situ hybridization
FLT3L: Fms related tyrosine kinase 3 ligand
FOXM1: Forkhead box protein M1
FPKM: Fragments per kilobase of transcript per million mapped reads
G-CSF: Granulocyte colony-stimulating factor
GO: Gene ontology
GSEA: Gene set enrichment analysis
HD: Healthy donor
HSPC: Hematopoietic stem and progenitor cells
IL-3: Interleukin 3
LAIP: Leukemia-associated immunophenotype
LSC: Leukemia stem cell
MACS: Magnetic-activated cell sorting
MKI67: Marker of proliferation Ki-67
MLL: Myeloid/lymphoid or mixed-lineage leukemia
NFKB: Nuclear factor kappa-light-chain-enhancer of activated B cells
NGS: Next-generation sequencing
NPM1: Nucleophosmin 1
PBS: Phosphate buffered saline
PCA: Principal component analysis
qPCR: Quantitative polymerase chain reaction
RPMI: Roswell park memorial institute
RT-PCR: Real-time polymerase chain reaction
SCF: Stem cell factor
shRNA: Short hairpin RNA
TNFA: Tumor necrosis factor alpha
TPO: Thrombopoietin
UPL: Universal Probe Library
UTR: Untranslated region
