# Supplementary material for: Blast cells surviving acute myeloid leukemia induction therapy are in cycle with a signature of FOXM1 activity
Source: BMC Cancer. 2021 Oct 28;21:1153. doi: 10.1186/s12885-021-08839-9 (PMC8554867; doi:10.1186/s12885-021-08839-9)
Supplement: Supplementary file 1 — Additional file 1: Table S1. Antibody panel used for flow cytometry. Table S6. Primary AML samples used for FOXM1 KD. [file 12885_2021_8839_MOESM1_ESM.docx]

**Table S1.** Antibody panel used for flow cytometry.

|  | | **FITC** | **PE** | **Per CP** | **PE-Cy7** | **APC** | **APC-H7** | **eFluor450** |
| --- | --- | --- | --- | --- | --- | --- | --- | --- |
| **TUBE 1** | ***Antigen***  *Clone*  *Supplier* | HLADR  L243  BioLeg | CD13  L138  BD | CD34  8G12  BD | CD117  104D2  eBio | CD33  P67.6  BioLeg | CD45  2D1  BD | CD3  OKT3  eBio |
| **TUBE 2** | ***Antigen***  *Clone*  *Supplier* | CD38  HB7  eBio | CD56  MY31  Tonbo | CD34  8G12  BD | CD117  104D2  eBio | CD33  P67.6  BioLeg | CD45  2D1  BD | CD7  M-T701  eBio |

Suppliers: BioLegend (San Diego, CA), BD Biosciences (Franklin Lakes, NJ),

eBioscience (Waltham, MA), Tonbo Biosciences (San Diego, CA).

**Table S6.** Primary AML samples used for *FOXM1* KD.

| BB^1^ | Age range | Sex | WHO (2016) | Karyotype |
| --- | --- | --- | --- | --- |
| 104 | 16-39 | F | AML with MLL-AF9 rearrangement | 46,XX, t(6;9;11)(p21;p22;q23) [variant of t(9;11)] |
| 106 | 60-79 | F | Therapy-related myeloid neoplasm (t-AML); MLL-AF6 | 46,XX, t(6;11)(q27;q23)[10] |
| 160 | 16-39 | F | AML with MLL-AF9 rearrangement | 46,XX, t(9;11)(p22;q23) |

^1^Biobank identifier.
