# Supplementary material for: Blast cells surviving acute myeloid leukemia induction therapy are in cycle with a signature of FOXM1 activity
Source: BMC Cancer. 2021 Oct 28;21:1153. doi: 10.1186/s12885-021-08839-9 (PMC8554867; doi:10.1186/s12885-021-08839-9)
Supplement: Supplementary file 3 — Additional file 3: Fig. S1. Flow cytometry gating strategy. Fig. S2. Leukemia associated immunophenotypes (LAIPs). Fig. S3. Gene set enrichment analysis (GSEA) and FOXM1 correlation analyses in post-chemotherapy blasts. Fig. S4. FOXM1 KD in AML cell lines. Fig. S5. FOXM1 knockdown in primary AML and normal CD34+ HSPCs. Fig. S6. FOXM1 inhibition in primary AML and normal CD34+ HSPCs. [file 12885_2021_8839_MOESM3_ESM.pdf]

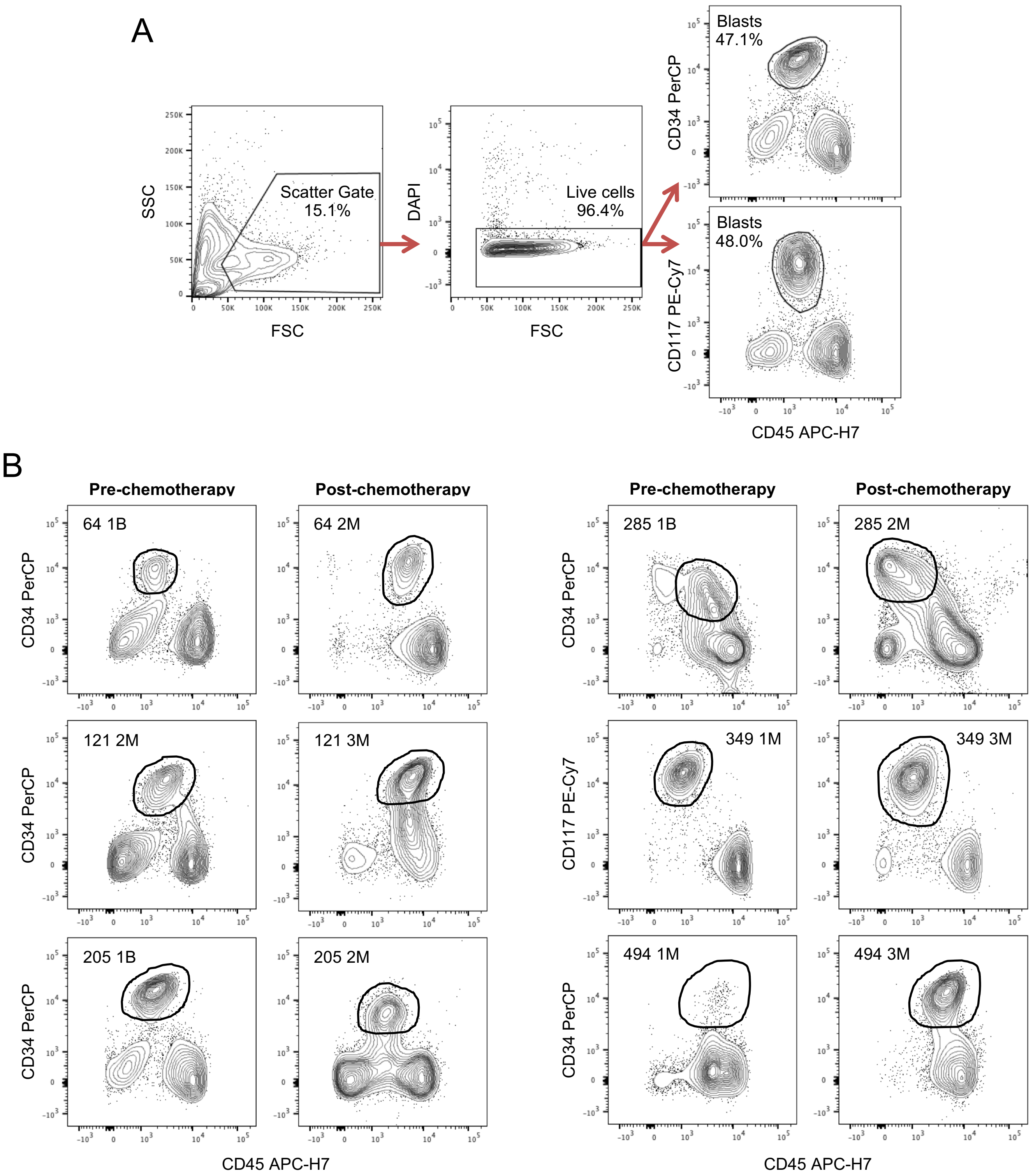

Figure S1. Flow cytometry gating strategy.

(A) Representative flow cytometry scatter plots showing the gating approach used to isolate blasts on the basis of scatter characteristics, DAPI negativity, CD45$^{low/int}$ expression and expression of either CD34 or CD117. (B) Flow cytometry scatter plots show gating strategy for the blast populations isolated from each sample.

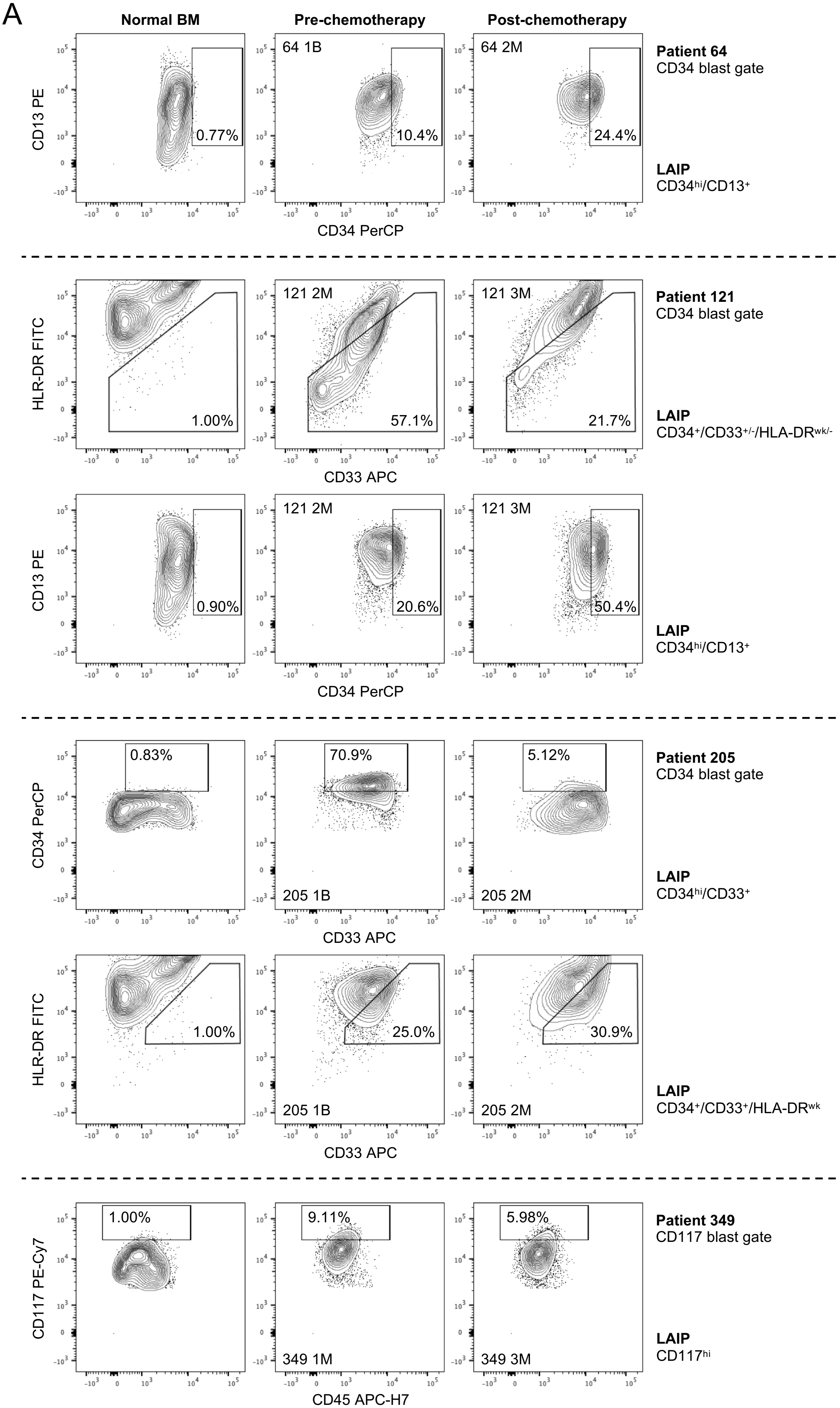
A
Normal BM
Pre-chemotherapy
Post-chemotherapy
64 1B
64 2M
0.77%
10.4%
24.4%
CD13 PE
CD34 PerCP
Patient 64
CD34 blast gate
LAIP
CD34hi/CD13+
121 2M
121 3M
1.00%
57.1%
21.7%
HLR-DR FITC
CD33 APC
Patient 121
CD34 blast gate
LAIP
CD34+/CD33+/-/HLA-DRwk/-
121 2M
121 3M
0.90%
20.6%
50.4%
CD13 PE
CD34 PerCP
LAIP
CD34hi/CD13+
0.83%
70.9%
5.12%
205 1B
205 2M
CD34 PerCP
CD33 APC
Patient 205
CD34 blast gate
LAIP
CD34hi/CD33+
1.00%
25.0%
30.9%
205 1B
205 2M
HLR-DR FITC
CD33 APC
LAIP
CD34+/CD33+/HLA-DRwk
1.00%
9.11%
5.98%
349 1M
349 3M
CD117 PE-Cy7
CD45 APC-H7
Patient 349
CD117 blast gate
LAIP
CD117hi

B

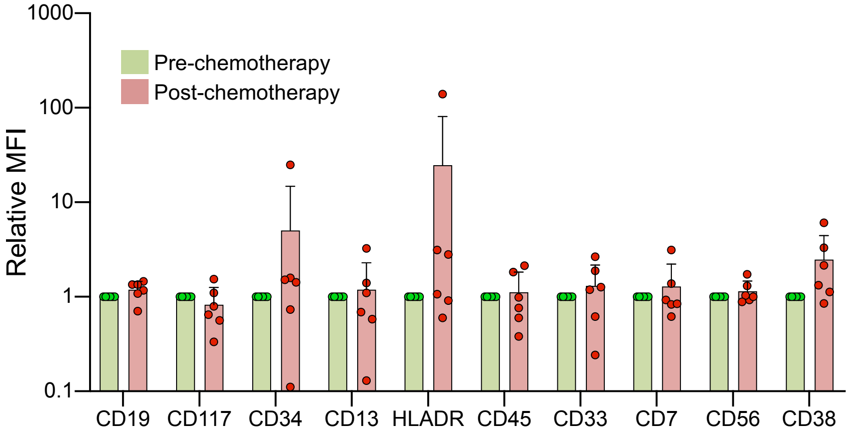

Figure S2. Leukemia associated immunophenotypes (LAIPs).

(A) Flow cytometry scatter plots showing the highest frequency leukemia associated immunophenotypes (LAIPs) identified in the indicated primary AML samples using a difference-from-normal gating strategy. (B) Mean±SD relative median fluorescence intensity (MFI) for the indicated cell surface markers on primary AML blasts pre- and post-chemotherapy (n=6). *P*=not significant by unpaired t-test for all comparisons.

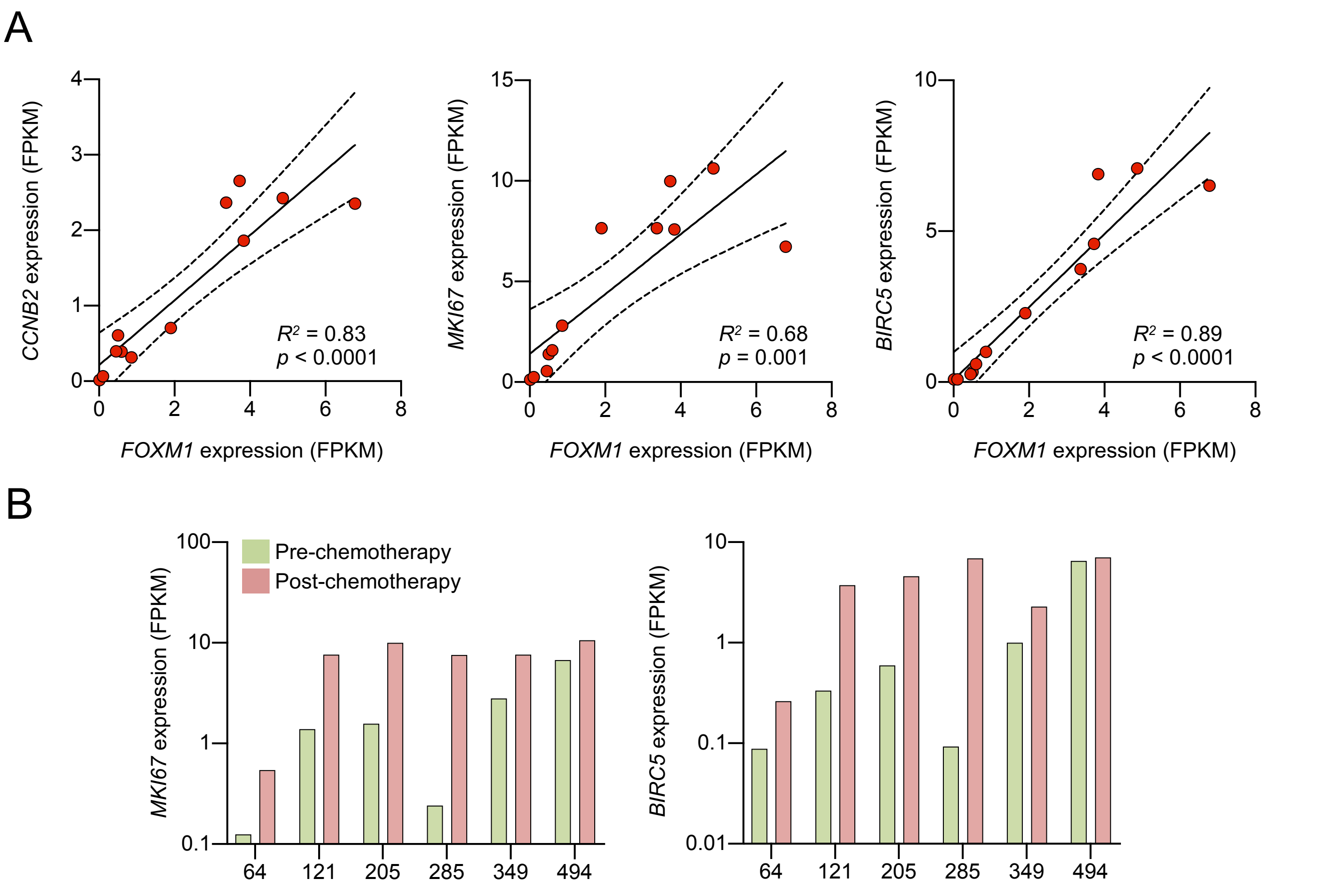

Figure S3. Gene set enrichment analysis (GSEA) and *FOXM1* correlation analyses in post-chemotherapy blasts.

(A) Scatter plots show correlations between *FOXM1* and the indicated cell cycle genes. *R* represents the Pearson product moment correlation coefficient. Dashed lines indicated 95% confidence intervals for the line of best fit. (B) Bar charts show expression of the indicated genes in all samples. FPKM, fragments per kilobase of transcript per million mapped reads.

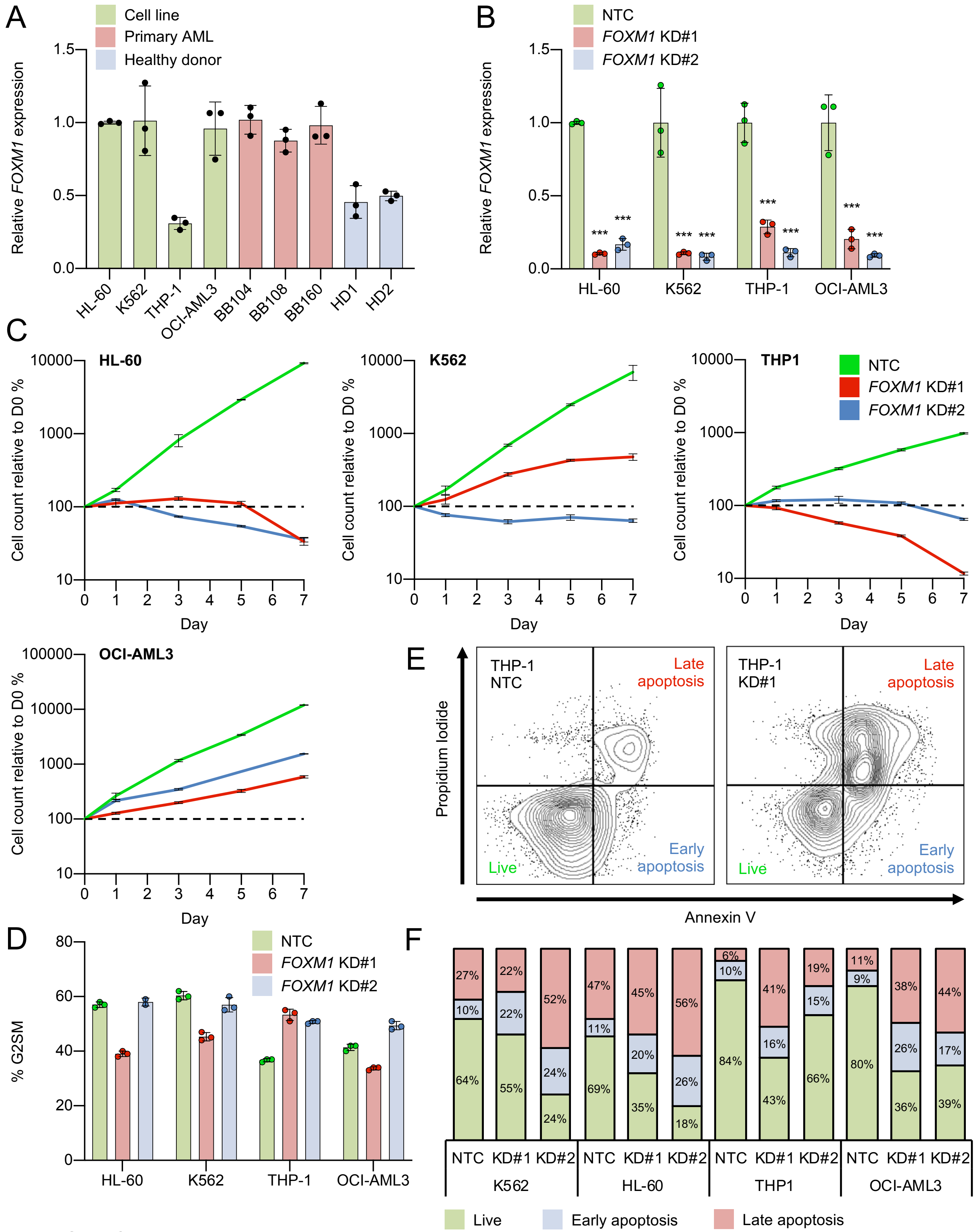

Figure S4. *FOXM1* KD in AML cell lines.

(A) Mean±SD (n=3) *FOXM1* expression relative to HL-60 cells by quantitative PCR in AML cell lines, primary AML and normal CD34⁺ HSPCs from healthy donors. (B) Mean±SD (n=3) *FOXM1* expression in KD versus control conditions for the indicated lines. *** P<0.001 (one-way ANOVA with Dunnett's multiple comparison test). (C) Graphs show mean±SD (n=3) cell count relative to Day 0 on the indicated day of culture for the indicated conditions. (D) Mean±SD (n=3) percentage of cells in G2SM in *FOXM1* KD versus control. (E) Representative flow cytometry scatter plots. (F) Proportion of live, early and late apoptotic cells for *FOXM1* KDs versus control for the indicated cell lines (n=3). NTC, non-targeting control; BB numbers indicate Biobank identifier.

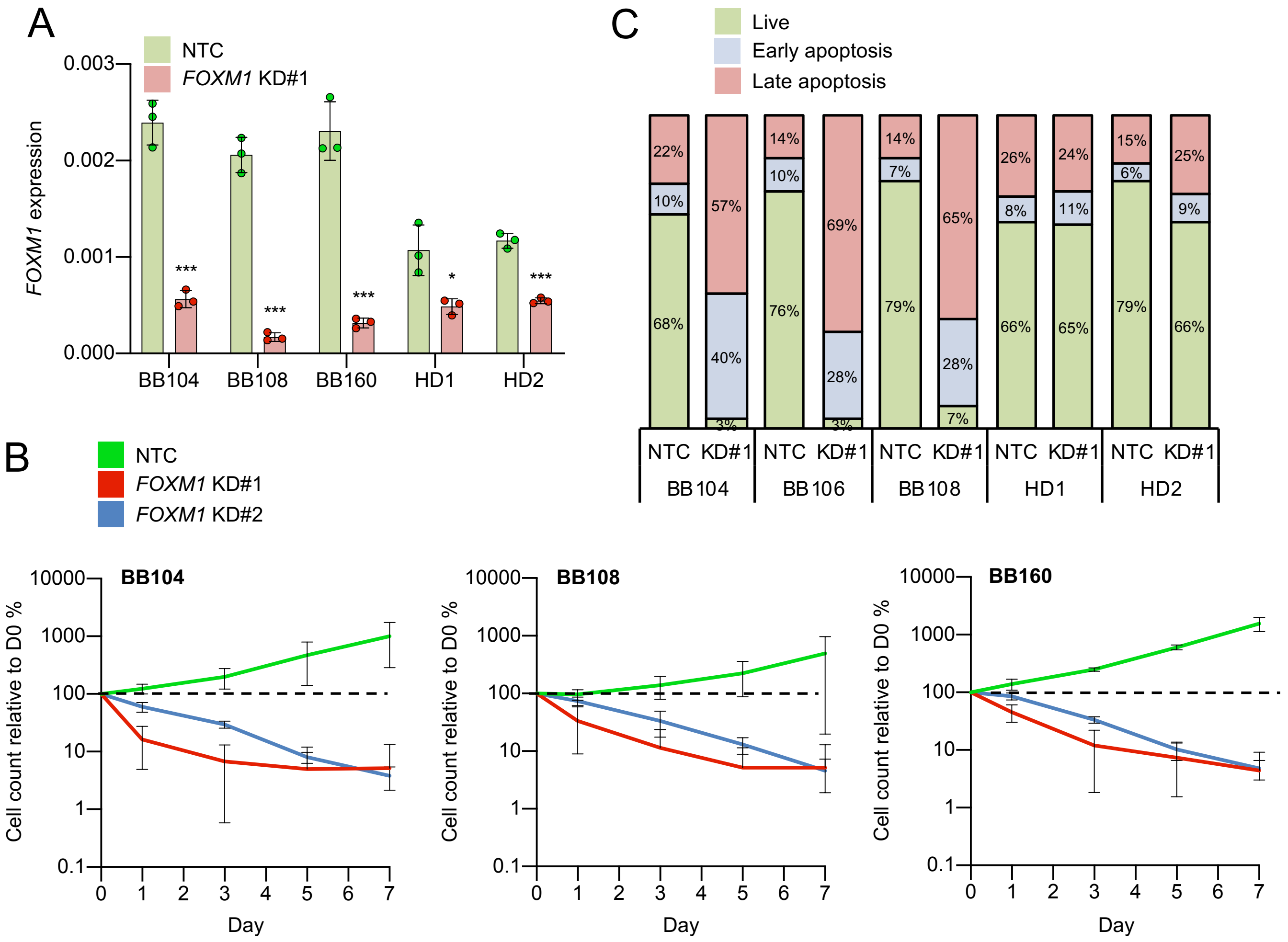

Figure S5. *FOXM1* knockdown in primary AML and normal CD34+ HSPCs..

(A) Mean±SD (n=3) *FOXM1* expression by quantitative PCR in the indicated conditions and the indicated primary AML and normal $CD34^+$ HSPC cell types. * *P*<0.05, *** *P*<0.001 by unpaired *t* test. (B) Graphs show mean±SD (n=3) cell count on the indicated day of culture for the indicated primary AML samples. (C) Proportion of live, early and late apoptotic cells for *FOXM1* KD versus control for the indicated primary AML and normal $CD34^+$ HSPCs (n=3 for primary AML, n=1 for normal CD34+ HSPCs); see Figure S4E for definitions. NTC, non-targeting control; BB numbers indicate Biobank identifier.

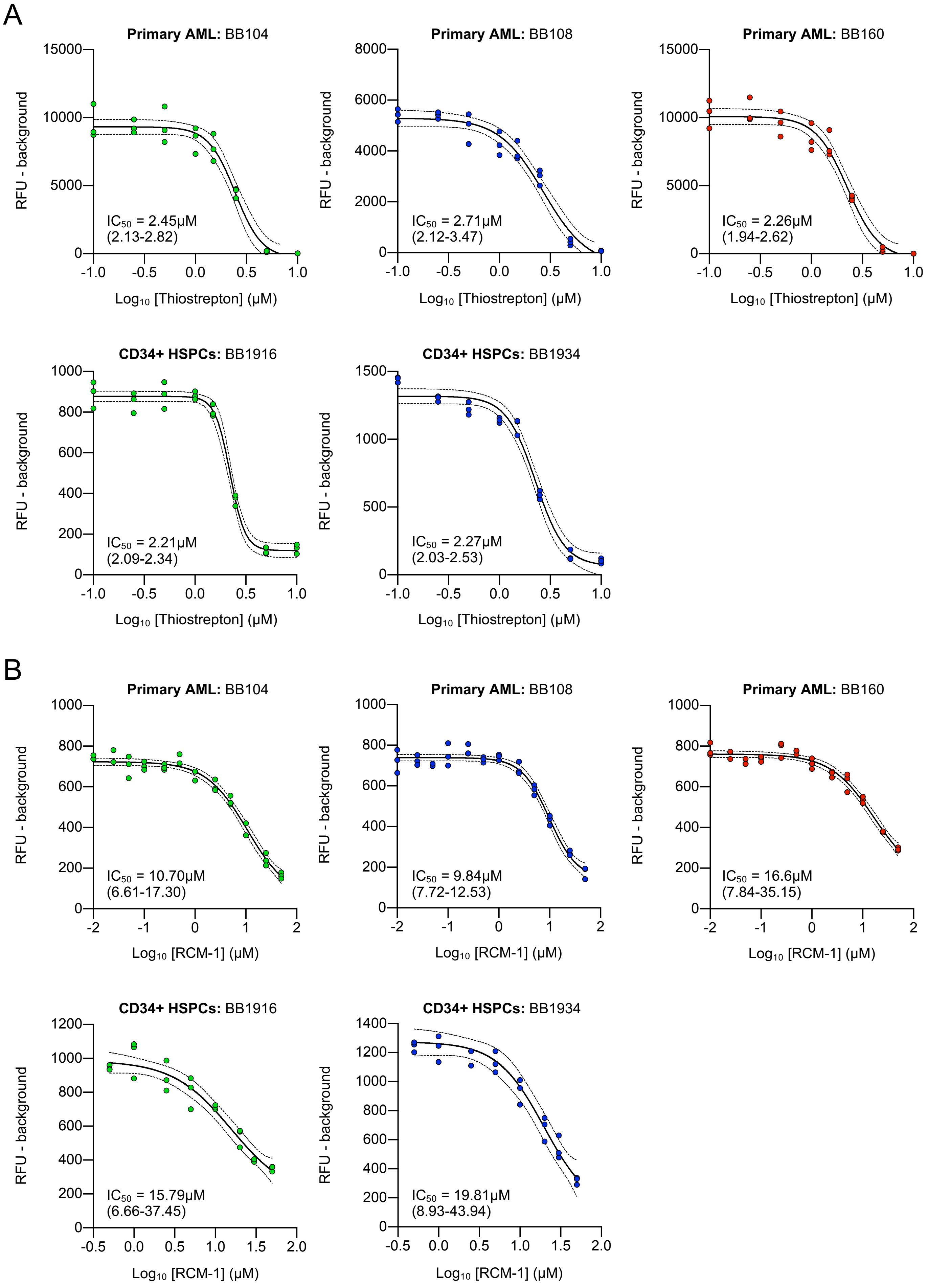
A
Primary AML: BB104
IC50 = 2.45μM
(2.13-2.82)
Primary AML: BB108
IC50 = 2.71μM
(2.12-3.47)
Primary AML: BB160
IC50 = 2.26μM
(1.94-2.62)
CD34+ HSPCs: BB1916
IC50 = 2.21μM
(2.09-2.34)
CD34+ HSPCs: BB1934
IC50 = 2.27μM
(2.03-2.53)
RFU - background
Log10 [Thiostrepton] (μM)
B
Primary AML: BB104
IC50 = 10.70μM
(6.61-17.30)
Primary AML: BB108
IC50 = 9.84μM
(7.72-12.53)
Primary AML: BB160
IC50 = 16.6μM
(7.84-35.15)
CD34+ HSPCs: BB1916
IC50 = 15.79μM
(6.66-37.45)
CD34+ HSPCs: BB1934
IC50 = 19.81μM
(8.93-43.94)
RFU - background
Log10 [RCM-1] (μM)

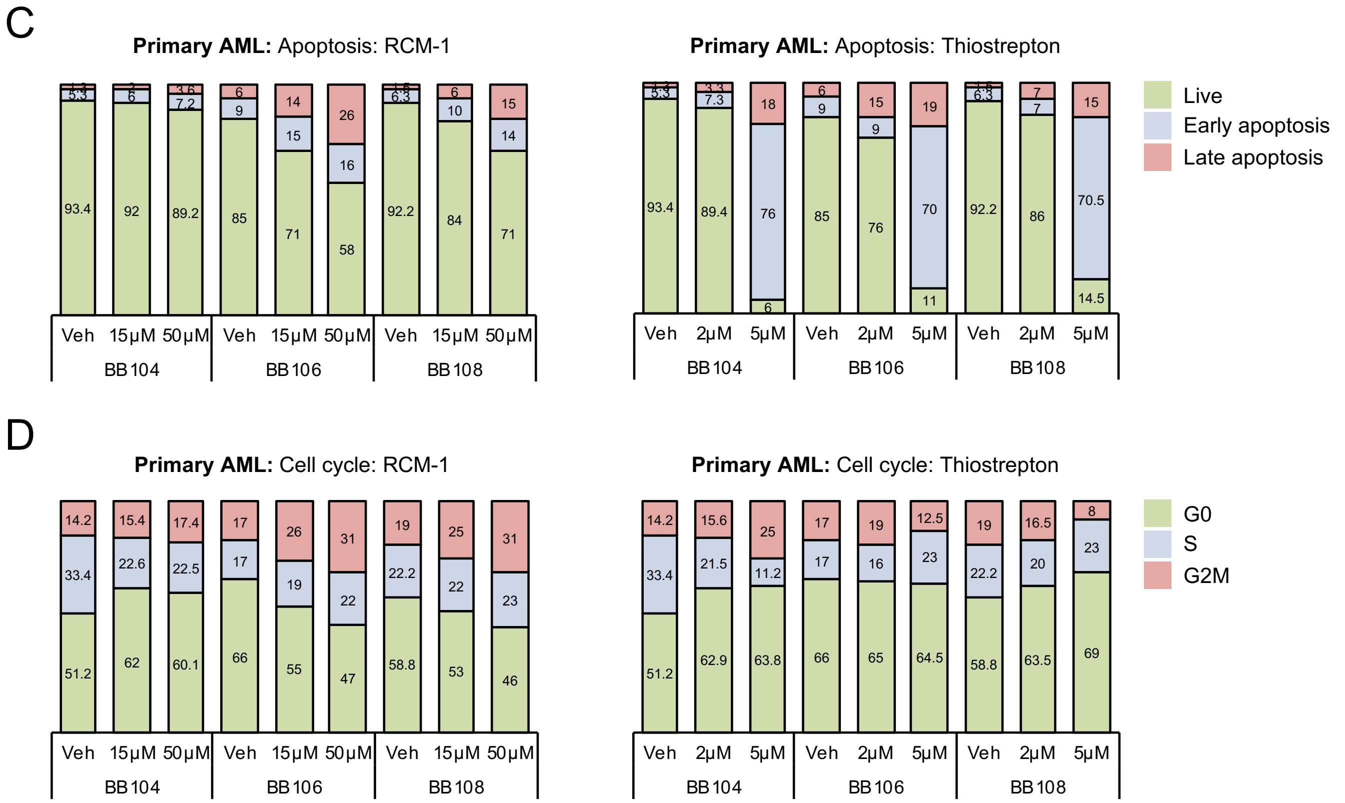

Figure S6. FOXM1 inhibition in primary AML and normal CD34+ HSPCs.

(A-B) Dose response curves for primary AML and normal CD34$^+$ HSPCs treated with Thiostrepton (A) or RCM-1 (B). IC_{50} shown with 95% CI in parentheses (n=3). RFU, relative fluorescence units. (C) Proportion of live, early and late apoptotic cells for RCM-1 (left) or Thiostrepton (right) versus vehicle for the indicated primary AML. (D) Proportion of cells in G0, S or G2M phase for RCM-1 (left) or Thiostrepton (right) versus vehicle for the indicated primary AML. BB numbers indicate Biobank identifier.
